# En Bloc Total Vertebrectomy of the Thoracic and Lumbar Spine

**DOI:** 10.3390/jcm13175312

**Published:** 2024-09-08

**Authors:** Eleonora Schneider, Marie-Christine Lutschounig, Jennifer Straub, Klemens Vertesich, Petra Krepler, Anna Rienmüller, Susanna Lang, Iris-Melanie Noebauer-Huhmann, Christoph Böhler, Reinhard Windhager

**Affiliations:** 1 Department of Orthopaedics and Trauma Surgery, Division of Orthopaedics, Medical University of Vienna, Waehringer Guertel 18-20, 1090 Vienna, Austria; marie-christine.lutschounig@meduniwien.ac.at (M.-C.L.); jennifer.straub@meduniwien.ac.at (J.S.); klemens.vertesich@meduniwien.ac.at (K.V.); anna.rienmueller@meduniwien.ac.at (A.R.); christoph.boehler@meduniwien.ac.at (C.B.); reinhard.windhager@meduniwien.ac.at (R.W.); 2 Department of Clinical Pathology, Medical University of Vienna, Waehringer Guertel 18-20, 1090 Vienna, Austria; susanna.lang@meduniwien.ac.at; 3 Department of Biomedical Imaging and Image-Guided Therapy, Medical University of Vienna, Waehringer Guertel 18-20, 1090 Vienna, Austria; iris.noebauer@meduniwien.ac.at

**Keywords:** complications, en bloc total vertebrectomy, long-term survival, neurological outcome, primary malignant bone tumours, sarcoma

## Abstract

**Background/Objectives**: We evaluated the outcomes of patients undergoing en bloc total vertebrectomy at our institution within the last three decades. The aim of our study was to analyse the oncological and neurological outcomes and the changes over time. **Methods**: We included 22 consecutive patients treated with a total vertebrectomy at our institution between January 1990 and December 2022. The standard follow-up protocol for sarcoma patients was performed. Early complications were defined as complications within the first three months postoperatively. Local recurrence was defined as the reoccurrence of a tumour at least four months after surgery. Adequate statistical methods were applied to evaluate the survival rates and the influence of potential risk factors. A *p*-value of <0.05 was considered statistically significant. **Results**: From 1990 to 2010, five total vertebrectomies were performed each decade, whereas twelve patients underwent the procedure in the period from 2010 to 2022. The mean follow-up period was 101.25 months (±112; 2–339). The one-, five- and ten-year overall survival rates were 91% (CI = (0.79; 1.00)), 59% (CI = (0.37; 0.81)) and 51% (CI = (0.27; 0.75)), respectively. For soft tissue tumours, the average overall survival was 6.2 years, whereas, for bone sarcomas, it was 13.6 years. None of the patients with wide surgical margins developed local recurrence. Complications necessitating revision procedures occurred in 54% of all cases. **Conclusions**: A total vertebrectomy is a highly demanding procedure, requiring accurate patient selection, meticulous preoperative planning and a highly collaborative interdisciplinary team. Adequate surgical treatment seems to be indispensable when aiming for curative treatment. Owing to the rarity of the indications, this procedure should be restricted to large tumour centres.

## 1. Introduction

In contrast to spinal metastases, which occur prevalently and account for up to 60% of secondary osseous lesions, primary malignant tumours of the spine are rare and account for fewer than 5% of all osseous neoplasms [1]. Due to their rarity, these tumours are prone to being overlooked or unrecognised and therefore are often diagnosed at a later stage, when the tumour has already invaded the adjacent structures. However, regarding the limbs, adequate surgical treatment seems to be indispensable when aiming for curative treatment. Following Enneking’s principles regarding the oncological approach, wide or at least marginal resection should be achieved [2]. The delicate adjacent anatomical structures in the spinal region preclude radical surgical approaches, but total vertebrectomy offers a viable treatment option in selected cases. It is mostly restricted to patients suffering from primary malignant spinal tumours, with a good overall health condition and no dissemination of the tumour, in order to achieve wide (i.e., histologically tumour-free) resection margins. In patients suffering from solitary spinal metastasis or lung cancer invading the spine with a promising life expectancy, the peri- and postoperative risks and expected complications need to be deliberately discussed. In 2021, Court et al. suggested that combined video-assisted thoracoscopic surgery (VATS) may be advantageous in spinal malignancies as it allows both anterolateral and posterior visualisation with the prone positioning of the patient [3]. However, despite the many technical and anaesthesiological advancements within the last few decades, this surgical technique is still demanding and associated with high morbidity and mortality. Nonetheless, due to the rarity of the condition and of patients under consideration for en bloc total vertebrectomy, the literature remains scarce. Thus, our study aimed to analyse the oncological and neurological outcomes and the self-reported quality of life of patients undergoing a total vertebrectomy (tVBR) within the last few decades at our institution and investigate the changes over time.

## 2. Materials and Methods

**Figure 1:**
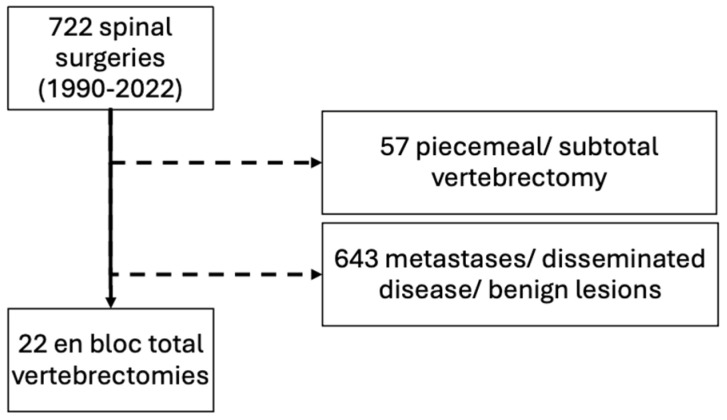
The flow chart shows the selection and eligibility criteria for inclusion in the present study.

Since 1952, our department has systematically documented tumour patient records in the Bone and Soft Tissue Tumour Registry. The corresponding institutional review board approved the study design and protocol (EK 642/2011), and informed consent was obtained from all patients operated on since 2011. Between January 1990 and December 2022, 722 surgical procedures on the mobile spine were performed, consisting of 79 with primary malignant tumours, and we could identify 22 consecutive patients (16 males (73%) and six females (27%), including five patients previously described) who were treated with total vertebrectomy at our institution [4]. All patients who underwent piecemeal or subtotal vertebral resection, as well as patients suffering from severe comorbidities or other circumstances impeding en bloc total vertebrectomy from the outset, were excluded from our study (Figure 1). Patient charts were screened for eligibility and follow-up records were evaluated. Patients included from 2011 onwards were continuously observed according to our standardised protocols. If still alive, patients undergoing surgery before 2011 were invited for follow-up. All histological specimens were analysed at the same corresponding pathological department over the whole study period.

### 2.1. Preoperative Planning

The tumour staging and preoperative planning followed our standardised protocol, including current conventional radiographs of the affected spinal region in two plains, magnetic resonance imaging (MRI) and computed tomography (CT). If necessary, angiography was also performed. In patients undergoing neoadjuvant therapy, radiographic imaging was repeated directly before surgery in order to adequately assess the resection margins. CT scans of the abdomen and chest, as well as, from 2010 onwards, positron emission tomography (PET) and scintigraphy, were performed to exclude secondary lesions. All cases were reviewed and discussed in our multidisciplinary tumour board, consisting of orthopaedic, oncological, radiotherapeutic, radiological, pathological and anaesthesiological specialists, on a weekly basis, also involving specialists from other departments (e.g., thoracic or plastic surgeons, but not exclusively), as required for individual cases. Seventeen patients (77%) were biopsied prior to surgery, including nine at an outside institution. Histological specimens, as well as previous radiographic data from patients undergoing biopsy at an outside institution, were reevaluated at our pathological and radiological departments. In the remaining five cases (23%), the patients were directly addressed with a total vertebrectomy, as three of them had definite and obvious results in the (PET) CT and MRI after a history of tumours (solitary spinal metastasis of a previously diagnosed and addressed primary bone tumour), and the remaining two patients underwent acute decompression surgery for neurological reasons at an outside institution and therefore representative histological specimens from prior surgery were available. The biopsy of one patient (5%), ultimately diagnosed with spindle cell sarcoma, was inconclusive and consequently had to be repeated.

### 2.2. Surgery

All surgeries were performed either by the head of our department or by trained members of our tumour and spine team. Between 1990 and 2010, we used an all-posterior tVBR technique, first described by Roy-Camille [5] and Tomita et al. [6]. This technique was considered feasible if at least one hemilamina was not infiltrated by the tumour [3]. It consisted of the contralateral posterior stabilisation of the spine at least two levels below and above the resected level(s), defining the resection margins; the resection of the adjacent ribs; and, finally, the careful rotation of the resected vertebrae around the spinal cord. The vertebral bodies were then replaced with either a mesh cage and bone graft or cement blocks/a bone graft. Finally, the ipsilateral side was stabilised. Since 2010, depending on the size, infiltration and location of the tumour, we have performed double-approach surgeries, using either an anterior–posterior or lateral–posterior approach to address the anterior part of the tumour. This shift in surgical approach was multifactorial. Firstly, it was driven by advancements in technical and anaesthesiological support in the operation room, as well as a transition towards more elaborate surgical techniques, making the double approach safer and allowing more comprehensive access and the removal of bone and surrounding structures. In most cases, the anterior approach is used for the preparation of the vessels; vertebral body resection, depending on the extension of the tumour; and replacement with a mash cage and bone graft or cement/bone graft. Finally, the surgery is completed with the resection of the posterior elements of the spine, the en bloc removal of the tumour and the stabilisation of the anterior and posterior columns of the spine, fixating at least two levels above and below the resection. Intraoperatively, the resection margins were histologically assessed using freshly frozen sections. According to Enneking’s classification, the resection margins were defined as intralesional, marginal or wide [2].

### 2.3. Follow-Up

At our department, the standard follow-up protocol for sarcoma patients consists of a clinical and radiographic examination of the tumour site and chest X-rays at four-monthly intervals for three years, six-monthly for a further three years and yearly thereafter. With increasing availability, thoracic/abdominal CT scans in the mentioned intervals and yearly bone scans were embedded into this algorithm, which now represents our institution’s standard of care. We subsequently applied advanced local imaging upon reasonable suspicion of a recurrent tumour in the X-ray. Early complications were defined as complications within the first three months postoperatively. Local recurrence was defined as the recurrence of a tumour at least four months after surgery. At the latest follow-up, a survey regarding post-interventional patients’ mobility and satisfaction, namely the Short Form-12 questionnaire (SF-12), was conducted [7].

### 2.4. Statistics

The analysis focused on the surgical, functional and oncological treatment outcomes of primary malignant tumours of the spine after a total vertebrectomy. As a primary endpoint, we defined the overall survival of patients undergoing en bloc total vertebrectomy at our department. The secondary outcome parameters were the comparison of survival according to the histological entity, tumour localisation, (neo-)adjuvant therapies, the decade of treatment and the patient’s age and sex. We abstained from sample size calculation due to the partly retrospective design of the study, as well as post hoc power analysis because those calculations cannot differentiate between statistical noise and clinically meaningful effects [8,9]. For all 22 patients, information on their sex, age, entity, location, approach and resection margin was available. This is presented in Table 1, giving a detailed case summary. As for missing data, such as the surgery time, we report the missing not at random (MNAR) nature of such data, as the surgical durations were only documented after 1997. Patients with missing data were not included in the analysis of the surgery duration. Furthermore, data on local recurrence, metastases and survival were available for all patients, given their thorough documentation in our institution’s tumour registry. Demographic (sex, age), pathological (site, resection margins, local recurrence, metastatic disease and death) and therapeutic parameters (surgery, (neo-)adjuvant therapy and function) were examined. Percentages were rounded up to whole numbers. Descriptive data (mean (±SD; range)) were reported for the entire patient cohort. The differences between means and proportions were tested with the Chi-square test for categorical variables and the unpaired *t*-test was used for continuous variables. Kaplan–Meier analysis was used to evaluate the survival rates. The log-rank test was used to compare the survival curves. All statistical tests were two-sided. A probability value of *p* ≤ 0.05 was considered statistically significant. Information was stratified by diagnosis, subsequent surgery, overall survival, complications and whether the patient had received (neo-)adjuvant therapy (chemotherapy, radiotherapy). Data were analysed using SPSS^®^ (version 26.0, SPSS Inc., Chicago, IL, USA).

## 3. Results

*Patient characteristics*. From 1990 to 2010, five total vertebrectomies (23%) were performed each decade, whereas twelve patients (55%) underwent the procedure from 2011 to 2022. The mean follow-up period was 101.3 months (±111.6; 2–339). The mean age at surgery was 44 years (±19.5; 6.3–73.6). The mean period between the date of the first presentation at our department and surgery was 2.3 months (±2.2; 0.0–6.9) on average. Two patients (9%) underwent spinal surgery at an outside institution before presentation at our department: one underwent an acute hemilaminectomy at Th2-4 due to paraparesis and the other patient presented to an outside institution with radicular pain caused by tumour-related neuro foraminal stenosis and underwent resection of the intraspinal tumour mass before its histological analysis. On their first visit to our department, 17 patients (77%) presented with pain and seven (32%) had neurological deficits. Two patients (9%) had fractures of the affected vertebra (Table 1).

*Histological entity*. Eight patients (36%) were diagnosed with chordoma and three with Ewing’s sarcoma (14%), while osteosarcoma, chondrosarcoma and haemangioendothelioma were found in two patients each (9%). Spindle cell sarcoma, plasmocytoma, myopericytoma, synovial sarcoma and malignant schwannoma were diagnosed in one patient each (5%).

*Localisation*. Eleven tumours (50%) were located in the thoracic spine, one (5%) in the thoracolumbar region (Th12-L1) and ten (46%) in the lumbar spine.

### 3.1. Surgical Results

*Approach*. Twelve patients (55%) were treated with solely posterior surgical access. From 2011 onwards, a two-staged total vertebrectomy with a ventral and dorsal surgical approach was performed in ten patients (46%). Three tumours treated with a double approach were located in the thoracic spine (30%), six in the lumbar spine (60%) and one in the thoracolumbar transition (10%). In six patients (27%), multisegmental resection was needed to achieve wide resection margins. To obtain optimal access for ventral reconstruction, partial or full rib resections had to be performed in ten patients (46%). In eight patients (36%), additional materials, such as a Vicryl mesh, Gore-Tex membrane or dura patch, were used.

**Figure 2:**
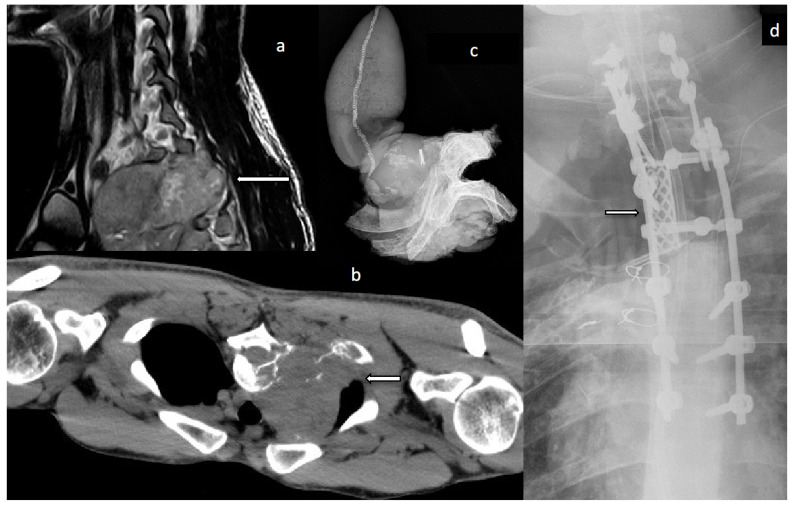
(**a**–**d**) male patient, 25 years, diagnosed with synovial sarcoma infiltrating the parietal pleura and the thoracic spine; (**a**) MRI scan in a sagittal view (arrow indicating intra- and extraosseous tumour extent); (**b**) CT scan in axial plane (arrow indicating intra- and extraosseous tumour extent); (**c**) intraoperative radiograph of histological specimen after en bloc resection of the vertebral body with adjacent ribs; (**d**) postoperative radiograph showing reconstruction with Harmscage (arrow) and dorsal stabilization C4-Th5.

*Duration*. From 1997, the surgery time was documented for all but one patient (N = 17; 77%). The mean duration of one (N = 12) or a multi-segmental (N = 5) total vertebrectomy was 9.8 h (±3.8; 4.4–15.3) and 14.2 h (±5.4; 8.7–22.1) per patient, respectively. The mean duration of hospitalisation after primary surgery was 42 days (±32; 14–141). For patients younger than 19 years at the time of surgery, the mean was 28.33 days, and for patients older than 60 years, it was 54.7 days, respectively. No significant difference regarding age, sex or the decade of treatment could be found. Thus, a trend towards a longer hospitalisation period with increasing age could be found. A representative case is shown in Figure 2.

### 3.2. Adjuvant Therapy

*Chemotherapy*. Eleven patients (50%) received chemotherapy, including nine neoadjuvant cases (Ewing’s sarcoma, osteosarcoma, chondrosarcoma, spindle cell sarcoma, chordoma, synovial sarcoma) and three treated postoperatively (plasmocytoma, osteosarcoma, synovial sarcoma).

*Radiotherapy*. In 15 cases (68%), radiotherapy was applied: nine neoadjuvant cases (spindle cell sarcoma, Ewing’s sarcoma, malignant schwannoma, myopericytoma, osteosarcoma, chordoma, synovial sarcoma) and seven after surgery (plasmocytoma, chordoma, synovial sarcoma).

### 3.3. Oncological Outcomes

Table 2 gives a detailed overview of the main oncological findings according to the histological entities and their survival.

**Figure 3:**
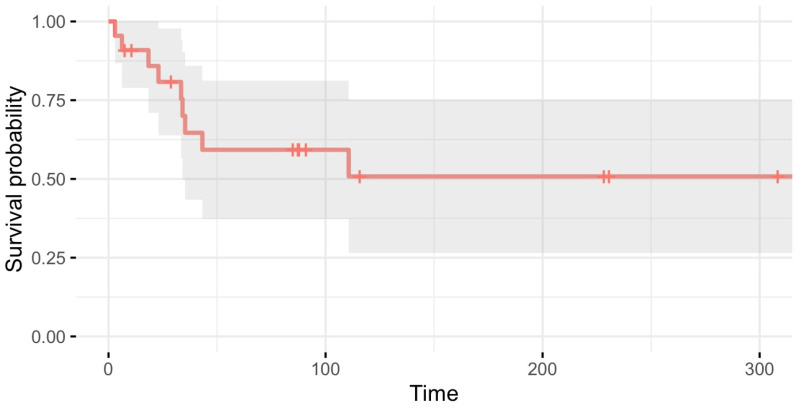
Overall survival. Kaplan–Meier survivorship curve for all patients (N = 22).

The one-, five-, ten- and 25-year overall survival (OS) rates were 91% (CI=(0.79; 1.00)), 59% (CI=(0.37; 0.81)), 51% (CI=(0.27; 0.75)) and 51% (CI=(0.27; 0.76)), respectively (Figure 3).

**Figure 4:**
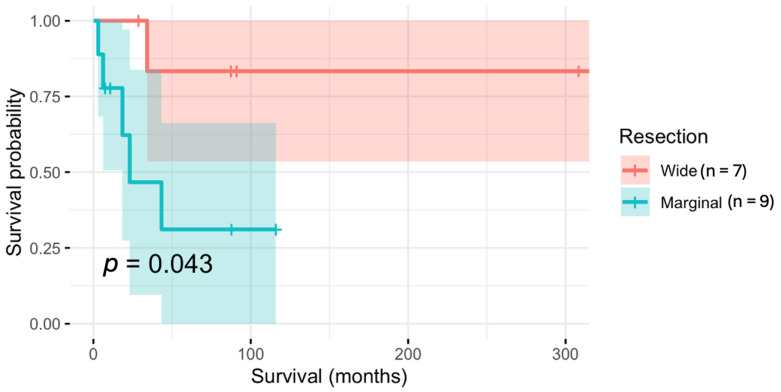
Survival probability according to resection margins. Kaplan–Meier estimates stratified by resection margin. Patients with wide resection margins had significantly better survival compared to patients with marginal resection margins. The p-value refers to the log-rank test.

*Resection Margins*. In 16 patients (73%), adequate resection margins could be achieved (wide resection in seven patients (32%), and marginal resection was achieved in nine cases (41%); Table 1). In six specimens (27%), the resection margins were intralesional; in two (9%), they were intentional. In one patient, the affected vertebra was fractured preoperatively and therefore dissemination was assumed. In a chordoma patient, tumour cells were found at the dorsal surface of the histological specimen, which was expected during surgical planning. Four patients (18%) had a positive resection margin, which was unexpected. The overall survival in patients with wide resection margins was significantly better when compared to those with marginal surgical margins (*p* = 0.043; Figure 4).

*Local Recurrence*. Overall, six patients (27%; five chordomas, one osteosarcoma) developed local recurrence after a mean time of 56.5 months (±59.1; 12–164). Four of them had marginal (66%) and two had intralesional (33%) resection margins. None of the patients with wide surgical margins (N = 7, 32%) developed local recurrence.

*Metastases*. Ten patients (46%) developed metastases after a mean time of 38.7 months (±52.3; 1.6–164.3). Three patients had wide (30%), two had marginal (20%) and four had intralesional (40%) resection margins. Four of the patients with metastasising tumours were diagnosed with chordoma (40%), three were diagnosed with Ewing’s sarcoma (30%) and one each (10%) were diagnosed with spindle cell sarcoma, osteosarcoma and plasmocytoma. In three patients (14%), a curative therapeutic approach was pursued, even though the addressed spinal lesion was a metastasis from a primary malignant bone tumour (OSA in the humerus and the femur, respectively; N = 2) or a chordoma in the knee joint (N = 1). Since all patients were young (<60 years) and in good health and spinal surgery was considered feasible, a total vertebrectomy was rated as the best treatment option regarding the patient’s survival.

*Survival*. Nine patients (41%) died after a mean time of 35.3 months (±31.9; 3.0–110.7); this included seven (78%) tumour-related deaths and two patients (22%) who died due to conditions not directly linked to their underlying disease. For bone sarcomas (N = 7; 32%), the mean survival period was 162.9 months (±154.8, 6–339). One patient had local recurrence (14%), and four cases developed metastases (57%). Two patients (29%) died due to bone sarcoma and another patient from causes not directly related. For soft tissue sarcomas (N = 6; 27%), the mean survival period was 74.8 months (±84.9; 3–231). None of them had local recurrence, one developed metastasis (17%) and two patients (33%) died from malignant disease. For chordoma (N = 8, 36%), the mean survival period was 71.2 months (±73.1, 11–229). Five patients developed local recurrence (63%), of whom three were treated with radiotherapy (60%). Three patients died due to their malignant disease (38%).

### 3.4. Neurological Outcomes

Four patients (18%) suffered from neurological impairment postoperatively (intercostal neuralgia Th9-11, sexual dysfunction, lesion of the left nerve plexus Th3 and sensory incomplete paraparesis from L1 on the right and Th12/L1 on the left side).

### 3.5. Patient-Reported Outcome Measures

Out of the13 patients who were alive, contact was successfully established with 10 patients (77%). A mean of 39.92 (±11.7; 21–52) on the physical score (PCS-12) could be reached. Regarding the mental score (MCS-12), the mean was 47.7 (±10.4; 30–62).

### 3.6. Complications

Early complications requiring revision surgery occurred in seven patients (32%) after a mean time of 1.1 months (±1; 0.0–3.0): six experienced wound healing disturbances (86%) and one experienced a complication related to the malpositioning of screws (14%). Regarding the complication rate, no significant causal relation to age, precedent radiotherapy, additional material and skin grafts could be drawn. Late complications followed by revision surgery occurred in eight patients (36%) after a mean time of 21.5 months (±20.4; 6.7–57.0). In six patients, material failure occurred (27%), requiring revision surgery after a mean time of 30.3 months (±22.5; 8–58.2). Dislocation of the material followed by revision surgery was seen in two patients (9%).

## 4. Discussion

In our consecutive series of total vertebrectomies with a follow-up period of eight years on average (range 1.6 months–28.3 years), we found an average overall survival period of 6.2 years for STS and 13.6 years for bone sarcomas, revealing better survival with wide resection margins and for bone sarcomas compared to soft tissue sarcomas. The mean survival of patients treated in our department is well in line with the literature. The mean overall survival for OSA was 67 months, that for chordoma was 71 months, that for STS was 75 months, that for CSA was 173 months and that for EWS was 220 months. None of the patients with wide resection margins developed local recurrence, but we could not find any significance regarding the development of metastases. The overall complication rate was 43%, including seven patients with mild complications (six wound healing deficits and one intercostal neuralgia), leaving a severe complication rate of 27%. The complication rate did not differ significantly over the decades as the learning curve progressed over time, but the cases became more complex and the number of multi-level resections increased. Although the methods of preoperative imaging changed from 2010 onwards, we could not find any significant impact on survival when comparing the subgroups of patients operated on before and after 2010. However, the cohort of patients diagnosed with chordoma and undergoing en bloc total vertebrectomy rose from two to six patients, which might have been linked to improved preoperative assessments, as well as the increased confidence in this surgical procedure, so that the combined risks even for semi-malignant tumours were more likely taken from 2010 onwards. Missenard et al. recently stated that, among others, diagnostic and especially therapeutic errors represent a significant loss of opportunity for patients suffering from spinal tumours, as the possibility of secondary salvage or rescue therapy is nearly non-existent. Surgery with positive resection margins is the most significant predictor of negative outcomes [10,11,12,13,14,15,16]. Araujo et al. published a case series of total vertebrectomies in 2018, reporting on 17 patients (19). The mean survival time after en bloc vertebrectomy was 23 months, differing for metastatic lesions (15 months) and localised disease (34 months). Overall, 77% of patients experienced intra- or postoperative complications, followed by revision surgery in 46%. Boriani et al. focused on the predictors of surgical complications following spinal en bloc resection by reviewing 220 cases [17]. In 45.5% of all cases, at least one complication occurred within the study period of 25 years, and the incidence of complications was stable throughout all years. The identified main risk factors were previous surgery or RTX, a double combined approach, incomplete intraoperative control of the haemodynamics, excessively short posterior fixation and a lack of anterior support. The decision-making process should consider the high morbidity rate against the positive impact of local tumour control and a better prognosis. Mody et al. reported achieving R0 resection in 89% of sarcoma patients and 85% of patients with metastatic lesions when applying the adopted en bloc resection [3]. Morbidity followed in 56% of all operated cases. Due to the long operating times, 9% of the patients suffered from pulmonary embolism and postoperative pneumonia. Court et al. reported on 33 video-assisted thoracoscopic en bloc vertebrectomies, including nine total vertebrectomies, addressing the previously reported risk factors. They achieved tumour-free resection margins in 85%, a complication rate of 55% and a median surgery time of 240 min [3]. In 2014, Mazel et al. focused on patients’ quality of life (QoL) after a total and partial vertebrectomy and showed that their long-term QoL after surgery was similar to that of the general population when comparing SF-36 data [18]. They concluded that the prolonged life expectancy justifies the aggressive surgery; furthermore, this type of surgical approach can also be successful at a functional level. As for all studies with a small sample size and retrospective design, our results need to be handled with great care. There are considerable limitations to this study, which primarily concern the retrospective, single-centre design and the low patient number itself, as this limits the prognostic value of our data and therefore implies the need for a critical interpretation of the deduced conclusions. The results may also be biased due to the long study period, as the treatment protocols and technical equipment have changed over the last few decades. Nonetheless, as for all rare entities and elaborate surgical techniques, large, prospective multi-centre studies are required in order to address these concerns.

## 5. Conclusions

En bloc total vertebrectomy is a highly demanding procedure requiring accurate patient selection, meticulous preoperative planning and a skilled, highly collaborative interdisciplinary team. Due to the rarity of tVBR, this procedure should be restricted to large tumour centres to provide optimal oncological and functional outcomes. Owing to the rarity of primary malignant tumours of the spine, multicentre studies should be performed to obtain a better understanding and optimised treatment options.

## Figures and Tables

**Table 1:** Detailed case summary; age given in years; M: male, F: female, CSA: chondrosarcoma, EWS: Ewing sarcoma, OSA: osteosarcoma, L: lumbar, Th: thoracic, Double: anterior and posterior approach; intrales.: intralesional, int. intra.: intentionally intralesional.

Case	Sex	Age	Entity	Location	Approach	Resection Margin
1	F	16.3	CSA	Th3	posterior	wide
2	M	36.0	Spindle Cell Sarcoma	Th6-8	posterior	wide
3	F	6.3	EWS	L3	posterior	wide
4	F	34.2	Schwannoma	Th10	posterior	marginal
5	M	18.3	EWS	L3	posterior	wide
6	M	34.5	Plasmocytoma	Th12	posterior	intrales.
7	F	47.0	Myopericytoma	Th9	posterior	intrales.
8	M	65.2	Chordoma	Th12	posterior	int. intra.
9	M	62.3	Chordoma	L3	posterior	intrales.
10	F	35.2	OSA	Th9	posterior	marginal
11	M	66.6	Chordoma	Th7-9	double	marginal
12	M	54.2	Chordoma	L3	double	marginal
13	M	14.9	OSA	L4	double	wide
14	M	41.5	Haemangioendothelioma	Th3	posterior	wide
15	M	44.5	Haemangioendothelioma	L1	posterior	intrales.
16	F	63.2	Chordoma	L2	double	marginal
17	M	73.6	CSA	Th12-L1	double	marginal
18	M	46.0	EWS	L3	double	marginal
19	M	59.8	Chordoma	L5	double	wide
20	M	56.5	Chordoma	Th11	double	int. intra.
21	M	25.4	Synovial Sarcoma	Th1-3	double	marginal
22	M	66.5	Chordoma	L3	double	marginal

**Table 2:** Oncological results for different entities in months; all values are given as mean (±SD; range); N: number of patients, EWS: Ewing sarcoma, CSA: chondrosarcoma, OSA: osteosarcoma, STS: soft tissue sarcoma, DOD: death of disease.

Entity	N	Follow-Up	Disease-Free Survival	Months to Metastasis	Months to Local Recurrence	DOD
Chordoma	8	70 (±74; 11–229)	41 (±57; 0–164)	50 (±77; 4–164)	62 (±64; 12–164)	3
EWS	3	213 (±184; 2–330)	43 (±50; 1–98)	43 (±50; 2–98)	—	1
CSA	2	173 (±236; 6–339)	171 (±237; 4–339)	—	—	0
OSA	2	67 (±34; 43–91)	56 (±49; 22–91)	22	29	1
STS	6	74 (±86; 3–231)	7 (±86; 0–231)	27	—	2
Plasmocytoma	1	111	9	9	—	0

## Data Availability

The raw data supporting the conclusions of this article will be made available by the authors on request.
